# CLDN4 as a Novel Diagnostic and Prognostic Biomarker and Its Association with Immune Infiltrates in Ovarian Cancer

**DOI:** 10.1155/2023/1075265

**Published:** 2023-04-04

**Authors:** Pan Hu, Li Lei, Ying Wang, Xue Tian, Xia Wei, Ni Jiang, Lubin Liu

**Affiliations:** ^1^Department of Obstetrics and Gynecology, Chongqing Health Center for Women and Children, Chongqing, China; ^2^Department of Obstetrics and Gynecology, Women and Children's Hospital of Chongqing Medical University, Chongqing, China; ^3^Department of Gynecology, Women and Children's Hospital of Xiushan County, Chongqing, China; ^4^People's Hospital of Kunming, Xishan District, Kunming, Yunnan, China

## Abstract

Ovarian cancer (OC) is the seventh most prevalent type of cancer in women and the second most common cause of cancer-related deaths in women worldwide. Because of the high rates of relapse, there is an immediate and pressing need for the discovery of innovative sensitive biomarkers for OC patients. Using TCGA and GSE26712 datasets, we were able to identify 17 survival-related DEGs in OC that had differential expression. CLDN4 was the gene that caught our attention the most out of the 17 important genes since its expression was much higher in OC samples than in nontumor samples. The findings of the ROC assays then confirmed the diagnostic utility of the test in screening OC specimens to differentiate them from nontumor specimens. Patients with high CLDN4 expression predicted a shorter overall survival (OS) and disease-specific survival (DSS) than those with low CLDN4 expression, according to clinical research. Patients with low CLDN4 expression predicted longer OS and DSS. Analysis using both univariate and multivariate techniques revealed that CLDN4 expression was an independent factor associated with a poor prognosis for OS and DSS. Based on multivariate analysis, the *C*-indexes and calibration plots of the nomogram suggested an effective predictive performance for OC patients. After that, we investigated whether or not there was a link between the infiltration of immune cells and the expression of the CLDN4 gene. We found that the expression of CLDN4 was positively associated with Th17 cells, NK CD56bright cells, while negatively associated with Th2 cells, pDC, and T helper cells. In the end, we carried out RT-PCR on our cohort and confirmed that the level of CLDN4 expression was noticeably elevated in OC specimens in comparison to nontumor tissues. The diagnostic usefulness of CLDN4 expression for OC was also validated by the findings of ROC tests. Thus, our findings revealed that CLDN4 may serve as a predictive biomarker in OC to assess both the clinical outcome of OC patients and the level of immune infiltration.

## 1. Introduction

Ovarian cancer (OC), which is one of the most prevalent but lethal forms of gynecological cancer, places a significant burden on the overall health of women all over the world [1]. Because of the lack of symptoms that are typically associated with OC until its more advanced phases, it has been given the nickname “the silent killer” [2, 3]. It is generally agreed that OC is a heterogeneous disease that includes at least five distinct subtypes, each of which possesses unique biological and molecular characteristics [4, 5]. Most people with OC were diagnosed at more advanced stages, which have a lower five-year survival rate (44% worldwide) [6]. This is because there are no evident symptoms in the early stages of the disease, despite the fact that many modern therapeutic approaches, like as surgery, immunotherapy, and targeted therapies, have been successful [7, 8]. The overall prognosis of patients with OC is not encouraging, and the risk of recurrence following treatment is high. As a result, they are unable to take advantage of the most beneficial treatment chances and do not utilize treatment strategies that are efficient, which leads to an unfavorable prognosis. Thus, we need to find better prognostic markers to better stratify patients and develop personalized therapeutic treatment strategies.

As more research on cancer development and metastasis has been carried out, there has been a corresponding surge in interest in tumor junctions (TJs) [9]. Several studies have indicated that the TJ plays a significant role in the advancement of cancer. As a constituent of tight junctions (TJs), the transmembrane protein CLDN, which has a size of around 20–27 kDa, is responsible for promoting cell-cell adhesion [10]. The CLDN molecule traverses the cell membrane four times on its way to the cytoplasm, where both its N- and C-termini are found. Because of their function as regulators of intercellular adhesion, CLDNs play significant roles in the process of carcinogenesis and have the potential to influence both the aggressive development and motility of tumors [11, 12]. In point of fact, there is mounting data suggesting that CLDN dysregulation is a characteristic shared by a wide variety of cancers, including gastric, lung, breast, ovarian, and colorectal cancer. Claudin-4, also known as CLDN4, is a key structural protein that is found in epithelial tight junctions [13]. It has a role in epithelial development, the maintenance of polarity, and considerable transport. Multiple research over the past few years have pointed to an essential function for CLDN4 in the development of various distinct types of cancer. On the other hand, very little information regarding the expression and function of CLDN4 was found in OC.

Tumor microenvironment (TME) was an intricate and dynamic multicellular ecosystem that included a variety of cell types, including immune cells, stromal cells, cancer cells, and other constituents [14]. Immune cells in the TME have long been recognized as a critical and core field of oncology inquiry [15]. These cells play an important part in the prognosis of cancer patients, as well as in immune evasion and treatment resistance. In terms of the release of cytokines and the recruitment of immune cells, the immunological microenvironment has an influence on the survival, proliferation, and migration of tumor cells [16–18]. Within this group, invading M2 macrophages plays a very significant role in the process. M2 macrophages develop from macrophages in the extraordinarily complex microenvironment of a tumor. These macrophages play a significant role in the regulation of tumor growth as well as invasion and metastasis. A deeper and more comprehensive understanding of endogenous antitumor immunity can be obtained through the examination of the density of immunocellular infiltration in tumor regions. In this study, our objective was to investigate the predictive usefulness of CLDN4 in OC as well as its connection with immune infiltration in OC.

## 2. Materials and Methods

### 2.1. Patients and Clinical Samples

All tissue samples, including those of the tumor as well as matched normal ovarian surface tissue, were taken from twenty ovarian cancer patients who had surgery at Chongqing Health Center for Women and Children between July 2020 and April 2022. None of the patients who were enrolled for this study had previously been treated with chemotherapy or radiation before their operations. Following the completion of the surgical excision, tumor specimens and the normal renal tissues that were close to the tumor were collected and frozen in liquid nitrogen until further use. The Research Ethics Committee of Chongqing Health Center for Women and Children gave their blessing to the current study before it was conducted. Consent to participate in the study was obtained from each individual patient.

### 2.2. RNA Extraction and Quantitative Real-Time PCR (qRT-PCR)

Through the use of TRIzol reagent (Invitrogen, Carlsbad, CA, USA), total RNA was isolated from frozen OC tissues. Using a PrimeScript RT Master Mix and one microgram of total RNA, high-quality cDNA was produced by the process of reverse transcription (Vazyme Biotech, Nanjing, China). Quantitative real-time PCR with a SYBR Premix Ex TaqII Kit (TaKaRa, Japan) was used to analyze the samples in triplicate to determine the amounts of mRNA. The primers used in this experiment were designed in-house (Tsingke, China). Internal quality check for the mRNA was performed with GAPDH. The 2′Ct technique was utilized for the purpose of determining the levels of CLDN4 expression. The PCR primer sequences were as follows: CLDN4 sense, 5′-TGGGGCTACAGGTAATGGG-3′ and reverse, 5′-GGTCTGCGAGGTGACAATGTT-3′; GAPDH reverse, 5′-ACAACTTTGGTATCGTGGAAGG-3′ and reverse, 5′-GCCATCACGCCACAGTTTC-3′.

### 2.3. Data Collection

The RNA-seq data were evaluated, and this included 427 individuals who had ovarian cancer that were obtained from TCGA database, as well as 88 samples of nondiseased ovarian tissue that were retrieved from the GTEx (Genotype-Tissue Expression) database. In addition, the RNA-Seq data of 185 OC patients were retrieved from the Gene Expression Omnibus (GEO) database. These data were based on the GPL96 platform and were included in the GSE26712 dataset.

### 2.4. Identification of DEGs in GSE26712 Datasets

Background errors were fixed, arrays were normalized, and differential expression analysis of 185 OC and 10 nontumor samples was performed with the help of the limma package of the R programming language. The threshold points for differentially expressed genes (DEGs) were determined to be samples that had an adjusted false discovery rate *P* that was less than 0.05 and a |log fold change (FC)| that was more than 2.

### 2.5. Survival Analysis

In order to study the relationship between gene expression and the overall survival (OS), disease-specific survival (DSS), and progress free interval (PFI) of OC patients, Kaplan-Meier plots were generated. A log-rank test was utilized in order to investigate the statistical significance of the correlation.

### 2.6. Identification of Independent Prognostic Parameters of OC

In order to validate the independent prognostic value of the gene signature and to identify independent prognostic parameters, univariate- and multivariate Cox regression analyses were performed in TCGA dataset on the prognostic gene signature and clinicopathological parameters. These analyses focused on validating the independent prognostic value of the gene signature. When *P* was less than 0.05, statistical significance was assumed. Only the parameters that had a *P* value that was less than 0.05 based on the univariate analysis were included in the subsequent multivariate Cox regression analysis.

### 2.7. Predictive Nomogram Construction and Validation

The independent prognostic indicators acquired from multivariate analysis were utilized to build nomograms, which individualized the expected survival probability for one, three, and five years. These nomograms were established on the basis of Cox regression models. It was decided to make use of the RMS software in order to generate nomograms that contained important clinical characteristics as well as calibration plots. The calibration curves were graphically evaluated by mapping the nomogram-predicted probability against the observed occurrences; the 45° line represented the best predictive values among all of the lines in the assessment. To evaluate the accuracy of the nomogram's discrimination, a concordance index, abbreviated as *C*-index, was utilized, and its value was determined using a bootstrap method with a total of 1,000 resamples. The *C*-index was utilized to make a comparison between the prediction accuracies of the nomogram and those of the individual prognostic parameters. In this particular research endeavor, all statistical tests were performed using two different sets of data, and the level of statistical significance was established at 0.05.

### 2.8. Function Enrichment Analysis of Differentially Expressed Genes between Groups with High CLDN4 Expression and Groups with CLDN4 Expression

In order to investigate the biological and molecular functions that CLDN4 played in OC, Gene Ontology (GO) and Kyoto Encyclopedia of Genes and Genomes (KEGG) were used. An investigation on the BP, CC, and MF that are related with CLDN4 was carried out using GO analysis. The Cluster Profiler program in R was used throughout each step of the three separate studies.

### 2.9. Infiltration of Immune Cells

The data from TCGA gene expression profile were used to quantify the infiltration of immune cells in tumor tissues using a method called ssGSEA (single-sample gene set enrichment analysis) [19]. The results of this study showed that there was an infiltration of 24 immune cells. SsGSEA calculates an enrichment score showing the degree to which genes in a certain gene set are coordinately up- or downregulated within a single sample. This score is based on the results of a collection of genes that have been studied. A gene's enrichment score is calculated by the ssGSEA by integrating the differences between the empirical cumulative distribution functions of its ranked genes. Genes are ranked according to the absolute expression they have in a given sample.

### 2.10. Statistical Analysis

All statistical analyses were performed in R (v3.6.2). The Wilcoxon signed-rank test was utilized for the analysis of paired samples, whilst the Wilcoxon rank-sum test was utilized for the analysis of unpaired samples. The receiver-operating characteristic (ROC) curve was used to analyze whether CLDN4 expression could be the diagnostic marker. In order to investigate the connection that exists between the expression of CLDN4 and the clinicopathological features, either the chi-square test or the Fisher exact test was carried out. A statistically significant *P* value was set at 0.05.

## 3. Results

### 3.1. Identification of Survival-Related DEGs in OC

**Figure 1:**
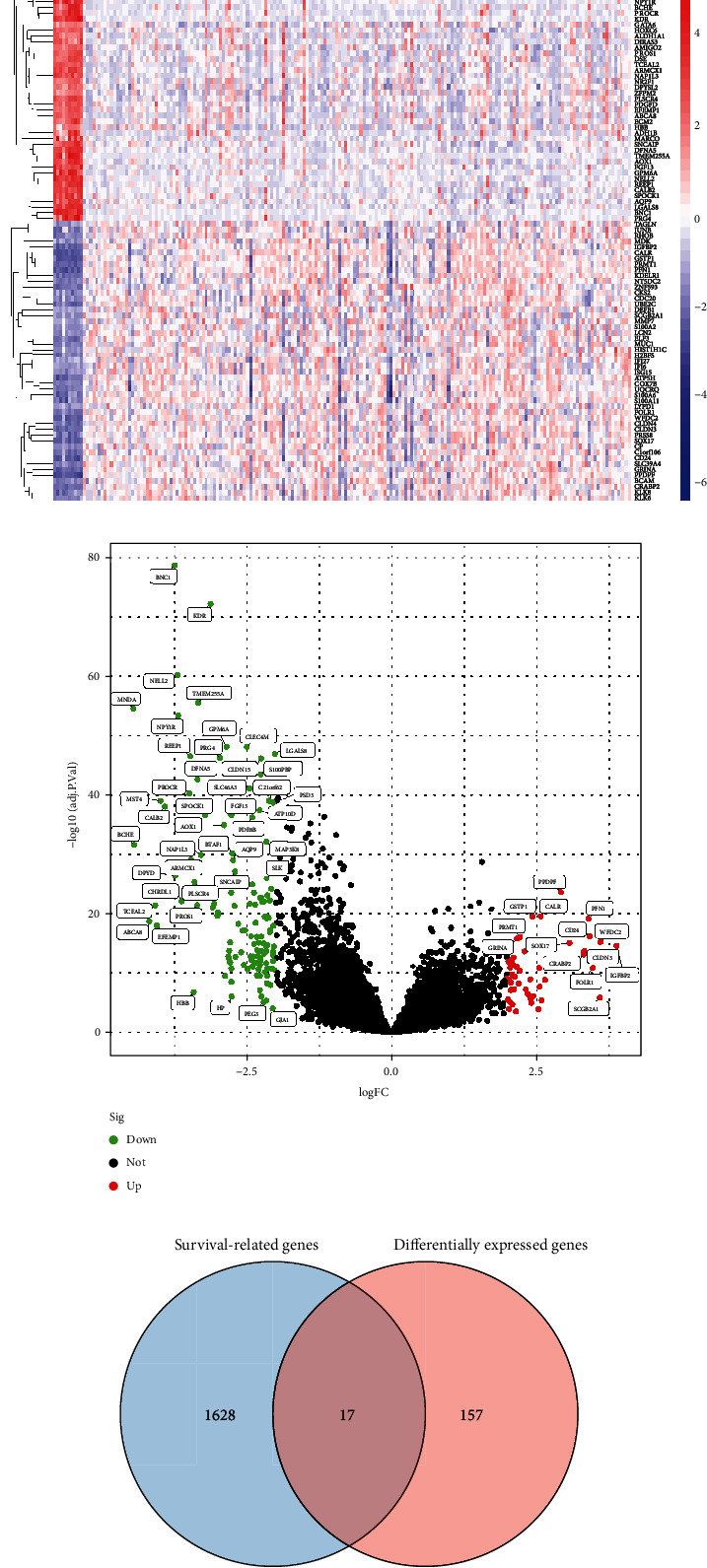
Identification of the survival-related DEGs between OC specimens and nontumor specimens. DEGs between OC tissues and nontumor specimens based on GSE26712 datasets were depicted in heat map and volcano plots, respectively (a, b). (c) The genes that are shared by the DEG GSE26712 datasets and the genes that are connected to survival according to TCGA datasets.

In this study, a retrospective analysis of the data was performed on a total of 175 OC samples and 10 nontumor samples taken from the GSE26712 datasets. A total of 174 differentially expressed genes (DEGs) were found, with 49 genes showing significant upregulation and 125 genes showing significant downregulation (Figures 1(a) and 1(b)). After that, we carried out survival study by making use of TCGA datasets, and we discovered 1645 genes in OC patients that are associated to survival.  Figure 1(c) illustrates the findings of a Venn diagram that confirmed 17 overlapping genes between 174 differentially expressed genes and 1645 genes related to survival (Figure 1(c)). CLDN4 was the primary focus of our research among the 17 genes that overlapped.

### 3.2. The Expression of CLDN4 in OC and Its Association with Clinical Factors

**Figure 2:**
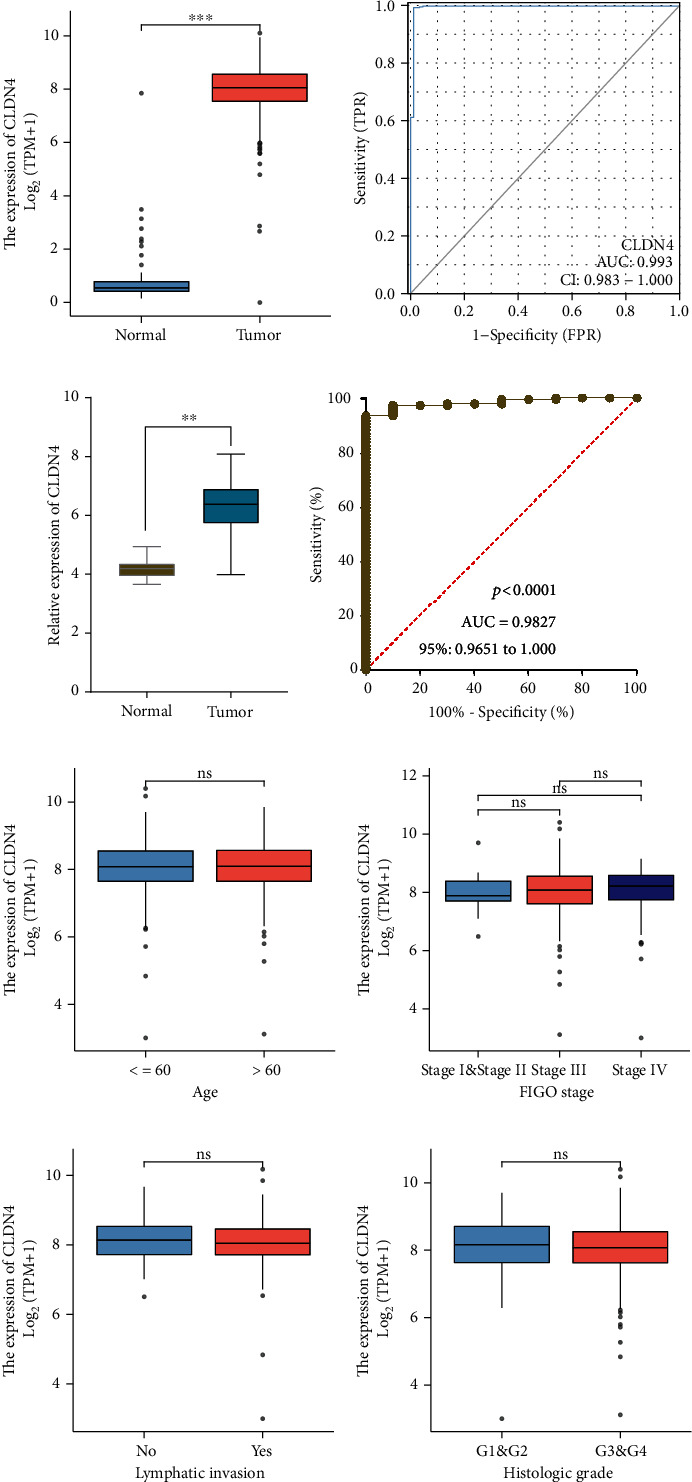
The expression of CLDN4 in OC and the clinical importance of its presence has been investigated. (a) When compared with nontumor specimens, OC were found to have a significantly higher level of CLDN4 overexpression. (b) ROC assays were utilized in order to provide evidence that CLDN4 expression have diagnostic value. (c) The expression of CLDN4 in OC as determined by the GSE26712 datasets. (d) ROC assays based on the data found in GSE26712. (e–h) Association between CLDN4 expression and clinicopathological parameters, such as age, FIGO stage, lymphatic invasion, and histologic grade.

First, we looked at the levels of CLDN4 expression in OC and found that it was significantly higher in OC samples than in nontumor samples. This data led us to conclude that CLDN4 is strongly upregulated in OC (Figure 2(a)). As a result, we conducted more research on the diagnostic potential of CLDN4. The findings of ROC testing revealed that CLDN4 efficiently distinguished OC specimens from normal specimens with an area under the ROC curves (AUC) of 0.993 (95% confidence interval [CI]: 0.983 to 1.000). These results are displayed in Figure 2(b). In addition, based on the findings from the GSE26712 datasets, we discovered that CLDN4 was substantially expressed in OC samples (Figure 2(c)). In addition, ROC testing proved the diagnostic utility of this method (Figure 2(d)). Using a pancancer investigation, we discovered that multiple different types of tumors had a dysregulated level of CLDN4, which suggests that this gene plays a significant role in the progression of cancers (Figure S1A and S1B). Following that, we investigated the potential relationships between CLDN4 expression and clinical factors. Despite this, we found that the expression of CLDN4 was not connected to a number of clinical variables, including age, FIGO stage, lymphatic invasion, and histologic grade (Figures 2(e)–2(h) and Table 1).

### 3.3. Survival Analysis of CLDN4 Expression in OC Patients

**Figure 3:**
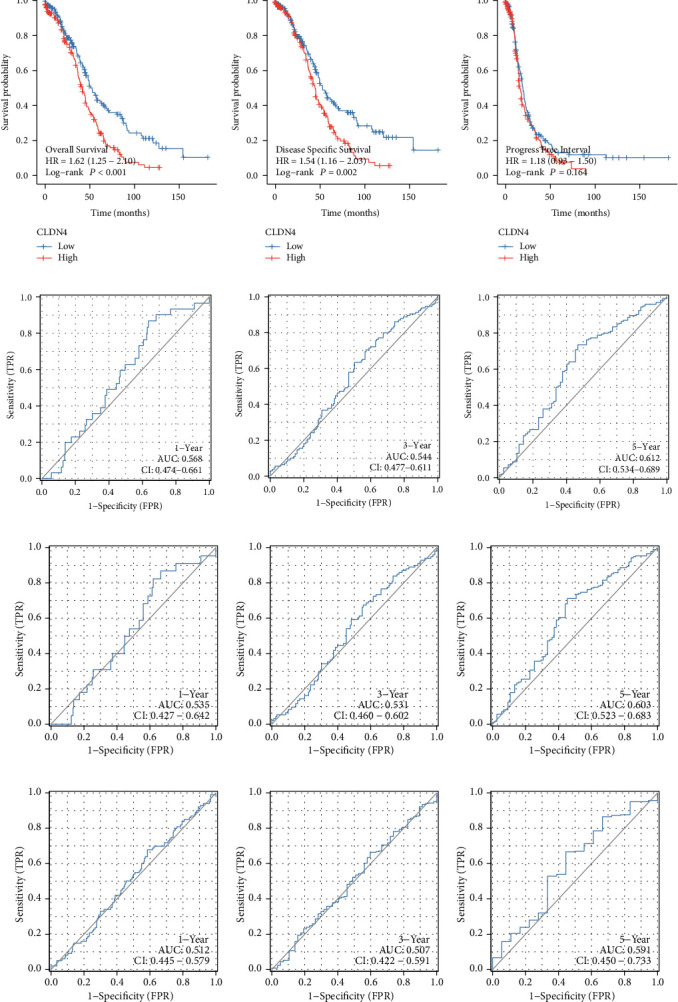
The survival study of CLDN4 expression in individuals diagnosed with OC. (a–c) Survival curves of overall survival, disease-specific survival, and progression-free survival for patients with OC who had high or low levels of CLDN4. The ROC curve was used to confirm that the expression of CLDN4 for (d) OS, (e) DSS, and (f) PFI is effective as a prediction tool.

**Figure 4:**
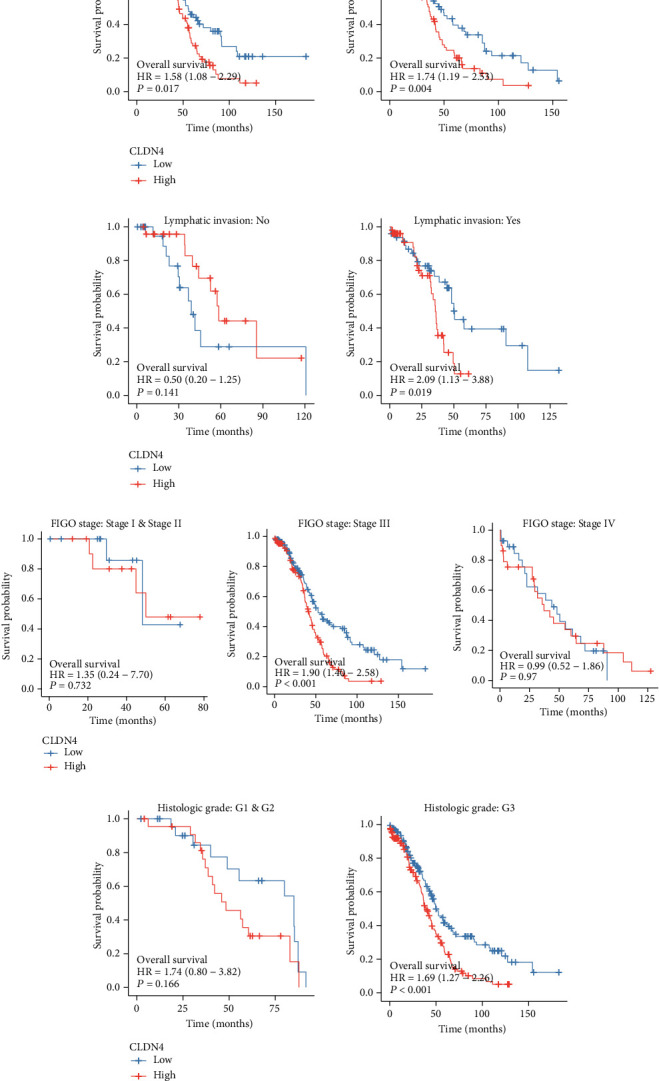
The survival analysis of CLDN4 expression in different subgroup of OC patients. (a) Age, (b) lymphatic invasion, (c) FIGO stage, and (d) histologic grade.

The next step consisted of conducting a survival study to investigate the predictive value of CLDN4 in OC patients. Patients who had high levels of CLDN4 expression predicted a shorter overall survival time and disease-free survival time than patients who had low levels of CLDN4 expression, as can be seen in Figures 3(a) and 3(b). On the other hand, we found no correlation between the expression of CLDN4 and the PFI of OC patients (Figure 3(c)). ROC curves illustrated the degree to which CLDN4 expression in TCGA cohort was able to accurately predict outcomes (Figures 3(d)–3(f)). In addition, we carried out subgroup analysis, which revealed that elevated CLDN4 expression demonstrated a significant correlation in both younger and older ovarian cancer patients (Figure 4(a)). CLDN4 expression was not linked with OS in patients with ovarian cancer who had nonlymphatic invasion (Figure 4(b)), early clinical stage (Figure 4(c)), and early histologic grade (Figure 4(d)). We performed univariate and multivariate analyses to demonstrate the predictive value of CLDN4 expression in OC patients. Importantly, both univariate and multivariate analyses showed that CLDN4 expression was an independent predictor associated with a poor prognosis for overall survival (Table 2) and disease-specific survival (Table 3). On the other hand, CLDN4 expression cannot be used to accurately forecast the PFI (Table 4).

### 3.4. Construction and Validation of a Nomogram Based on the CLDN4 Expression

**Figure 5:**
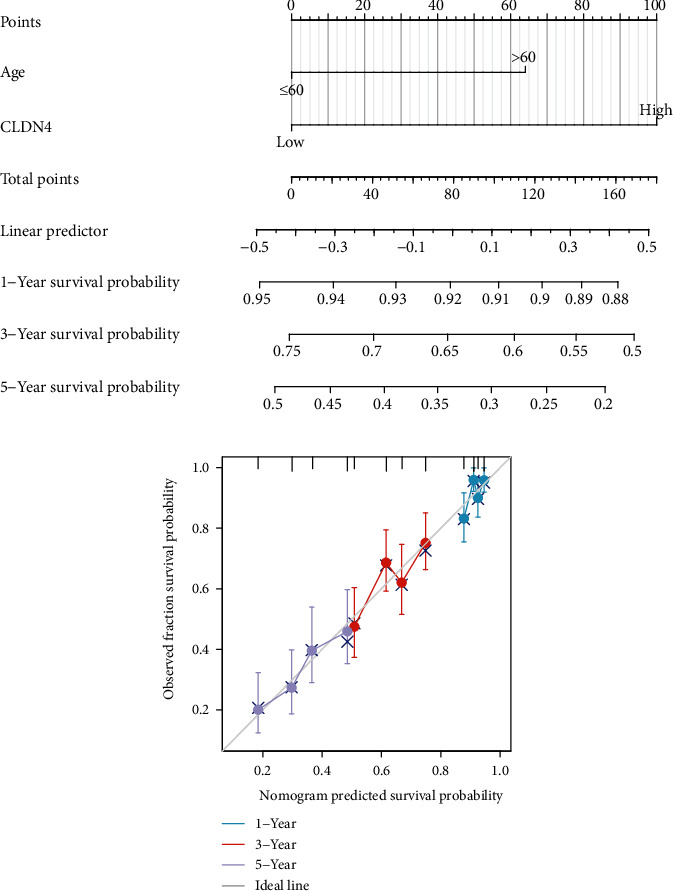
A quantitative method to forecast the probability of OC patients surviving one, three, or five years after their diagnosis. (a) A nomogram that estimates the likelihood that OC patients will be alive at 1, 3, and 5 years after diagnosis. (b) The calibration plots of the nomogram, which are used to forecast the likelihood of having OS at 1, 3, and 5 years.

In order to give a quantitative method for predicting the outcome of patients with OC, a nomogram was constructed using CLDN4 in conjunction with independent clinical risk indicators (Figure 5(a)). A point scale was utilized in the construction of the nomogram that was based on the multivariate Cox analysis. The variables were each given a certain number of points depending on the scale. The total number of points that were given to each variable was recalculated to fall within the range of one to one hundred. The sum of the points earned across all of the variables was then used as the basis for the final score. Drawing a vertical line immediately downward from the total point axis to the outcome axis allowed for the calculation of the chance of survival in OC patients at 1, 3, and 5 years. We also performed an analysis of the nomogram's ability to make correct predictions, and the findings showed that the *C*-index of the model was 0.584 (CI: 0.562-0.606), which indicated that the nomogram's ability to make accurate predictions is approximately accurate to a modest degree. The bias-corrected line in the calibration plot was employed to be close to the ideal curve, which was the line at 45 degrees, which showed that the forecast and the observation were in close agreement with one another (Figure 5(b)).

### 3.5. Functional Enrichment Analysis

**Figure 6:**
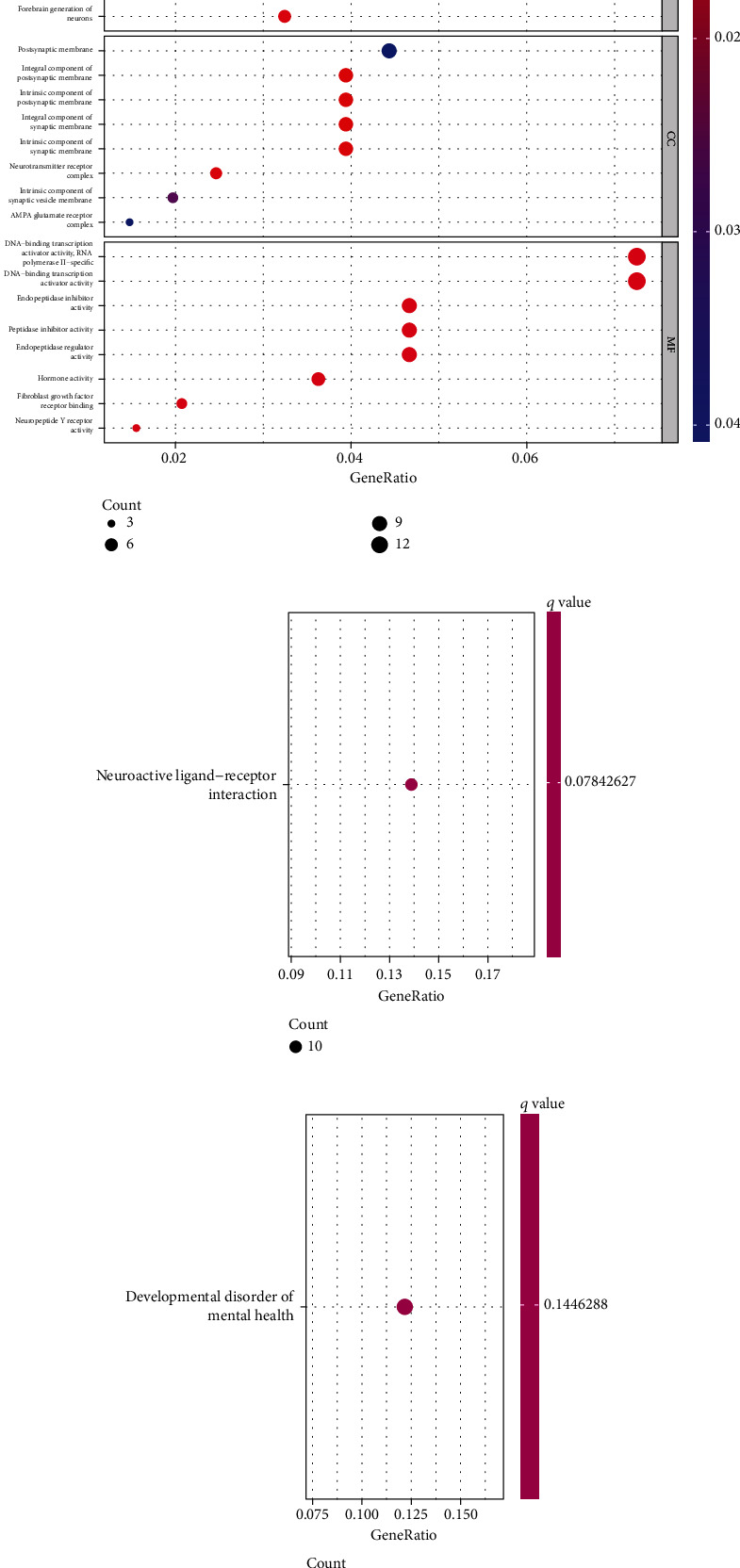
Functional bioassay. (a) GO functional analysis. (b) KEGG functional analysis. (c) DO functional analysis.

A total of 224 DEGs were discovered. After that, we carried out GO analysis with the help of 224 DEGs. As shown in Figure 6(a), we found that 224 DEGs were mainly enriched in regulation of ERK1 and ERK2 cascade, antimicrobial humoral response, sensory organ morphogenesis, postsynaptic membrane, integral component of postsynaptic membrane, endopeptidase inhibitor activity, peptidase inhibitor activity, and endopeptidase regulator activity. In addition, the results of KEGG revealed that the 224 DEGs were associated with neuroactive ligand-receptor interaction (Figure 6(b)). In order to learn more about the function of DEGs, enrichment analysis of DO pathways was carried out. According to the findings, the majority of the disorders that were enriched by DEGs were related to developmental disorder of mental health (Figure 6(c)). Our findings suggested that CLDN4 may be involved in the progression of several tumors.

### 3.6. The Association between CLDN4 Expression and Immune Cell Infiltration

**Figure 7:**
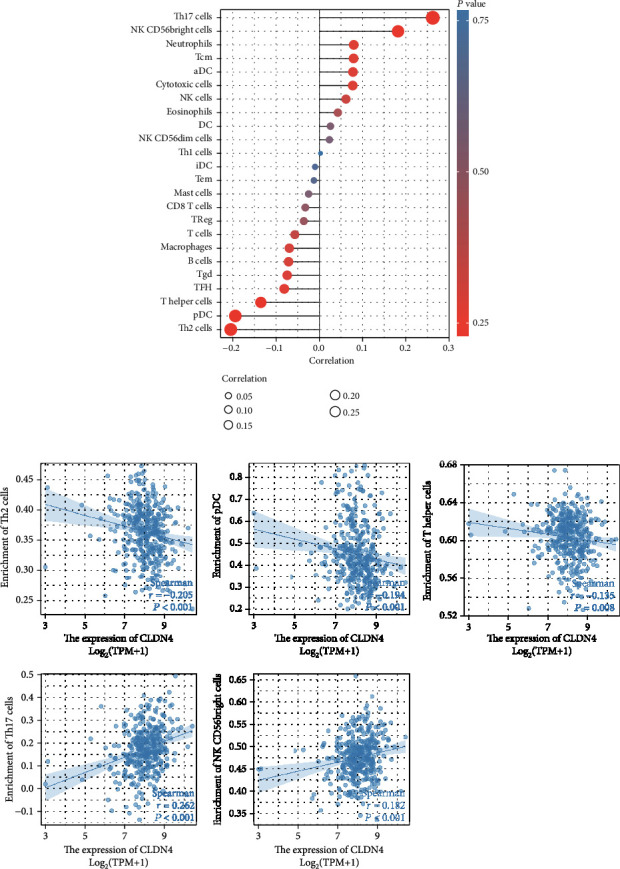
(a, b) Relationships between CLDN4 expression and infiltrating immune cells in OC.

Then, we explored the correlation between immune infiltration and CLDN4 expression. As shown in Figures 7(a) and 7(b), we found that the expression of CLDN4 was positively associated with Th17 cells and NK CD56bright cells, while negatively associated with Th2 cells, pDC, and T helper cells.

### 3.7. The Confirmation of CLDN4 Expression and Its Diagnostic in Our Cohort

**Figure 8:**
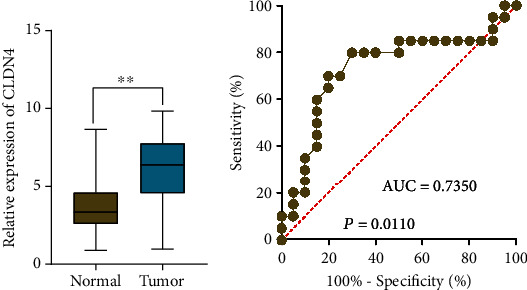
The level of expression of CLDN4 in OC in our cohort and the diagnostic value of this gene. (a) RT-PCR to analyze the expression of CLDN4 in OC and nontumor specimens. (b) The diagnostic usefulness of CLDN4 expression in screening OC specimens and differentiating them from nontumor specimens was validated by ROC analysis.

We used RT-PCR to investigate the level of CLDN4 expression in our sample population so that we could validate our previous findings. As can be seen in Figure 8(a), we discovered that the level of CLDN4 expression was noticeably higher in OC specimens in comparison to nontumor tissues. Following that, an investigation into the diagnostic utility of CLDN4 for OC patients was carried out. The ROC assays revealed that increased CLDN4 expression had an AUC value of 0.735 for OC (Figure 8(b)).

## 4. Discussion

The mortality rate from ovarian cancer, which already has the second highest rate among gynecological malignancies, is on the rise in China, but the prevalence of the disease is decreasing [20, 21]. It is difficult to detect in its early stages; thus, the majority of individuals are diagnosed when the disease has already progressed significantly [22, 23]. Even though there have been significant advancements in the treatment of OC, including chemotherapy, radiation, surgery, and targeted therapies, the 5-year overall survival rate for individuals with advanced OC is only about 30% [24, 25]. Thus, it is necessary to investigate the potential biomarkers related to the fundamental mechanisms of OC progression.

In recent years, a number of studies have indicated that an improper control of CLDN4 played a role in the evolution of a number of different cancers. For example, Hao and colleagues found that the expression of CLDN4 was abnormally increased in acute myeloid leukemia cells. In acute myeloid leukemia cells, inhibiting the expression of CLDN4 led to a significant reduction in cell proliferation as well as an increase in the rate of apoptosis. In addition, we discovered that inhibiting the expression of CLDN4 mRNA results in a suppression of the activation of AKT and ERK1/2. This suppression was achieved by knocking down CLDN4. Most notably, activating the AKT branch with SC79 partially counteracted the effects of CLDN4 knockdown on the suppression of cell survival. We also discovered that a higher expression of CLDN4 is associated with poorer survival and is an independent indication of shorter disease-free survival (DFS) in patients with acute myeloid leukemia [26]. According to the findings of Luo and colleagues, the expression of CLDN4 was much lower in gastric cancer tissues and cell lines when compared to nearby normal tissues or stomach epithelial cells. The silencing of CLDN4 led to a rise in the degree to which PI3K and Akt were phosphorylated, as well as in the proliferation, migration, invasion, and tumorigenesis of GC cells. Concurrently, apoptosis and the sensitivity of GC cells to chemotherapy were decreased. In conclusion, CLDN4 may play a critical role in improving the sensitivity of GC cells to chemotherapy and reducing the rate of GC cell proliferation by inactivating the PI3K/Akt signaling pathway. This may be achieved by inhibiting the activity of PI3K [27]. Jie et al. demonstrated that ELFN1-AS1 speeds up cell proliferation, invasion, and migration in ovarian cancer by modulating the miR-497-3p/CLDN4 axis. This finding suggests that CLDN4 acts as a tumor promotor in ovarian cancer. On the other hand, very little is known about the clinical importance of CLDN4 in OC. In this particular investigation, we discovered that OC specimens exhibited a markedly elevated level of CLDN4 expression. It was determined through survival assays that a high level of CLDN4 expression was related with a bad prognosis. Importantly, the results of the multivariate analysis suggested that the expression of CLDN4 was an independent factor associated with a poor prognosis for OS and DSS. Based on our findings, CLDN4 may serve as an innovative diagnostic as well as prognostic biomarker for patients with OC. In addition, our findings suggested that CLDN4 was highly expressed and predicted a poor prognosis. Thus, targeting CLDN4 may improve the clinical prognosis of OC patients.

The interaction between the TME and cancer cells is quite intricate, and the TME has strong ties to tumor cell proliferation, apoptosis, and the spread of the cancer to other organs [28, 29]. It has been hypothesized that the immune cells that make up healthy tissue, neighboring tissue, and malignant tissue are structurally distinct from one another in basic ways. It has been proven that the intrinsic mechanisms that contribute to immunotherapy resistance include the expression of particular genes and pathways in tumor cells. These genes and pathways have the ability to block the invasion or activity of immune cells in the TME. TME has been found to have a dual function in both the development of tumors and their initial appearance, according to a significant number of studies. Alterations to the TME have the potential to not only encourage the normalization of tumor cells but also to encourage tumor growth, invasion, and metastasis. B cells have been shown in a variety of studies to perform an anticancer role, either by directly interacting with tumor cells or by supporting in the operation of other immune functions. Treg cells are generally responsible for suppressing antitumor immunity, whereas CD8+ T cells are the primary antitumor effector cells [30]. During the course of this investigation, we came to the conclusion that the expression of CLDN4 was inversely linked with Th2 cells, pDC, and T helper cells, while it was positively associated with Th17 cells and NK CD56bright cells. Our findings suggested that CLDN4 was intimately connected to the invasion of immune cells and possesses significant potential as a therapeutic target in the treatment of cancer.

This study had certain shortcomings that need to be addressed. First, the predictive and prognostic usefulness of CLDN4 for the immune system needs to be verified in a larger number of OC patients who come from multiple real-world multicenters. Second, additional preclinical and clinical research is required to determine whether or not OC patients who have greater CLDN4 levels are more responsive to immune checkpoint inhibitors. Third, additional research, both experimental and clinical, is required to investigate potential techniques for enhancing immune function while minimizing the effects of an inhibitive milieu by focusing on CLDN4.

## 5. Conclusion

In the current investigation, we provide evidence that there was a connection between CLDN4 and OC. The results of this research showed that CLDN4 was an important gene in OC that has the potential to act as a predictive biomarker. Additionally, the researchers found that the expression of CLDN4 might be utilized to analyze immune infiltration in OC patients. To evaluate the accuracy of these predictors, however, additional research and experiments are required because the sample sizes were too small, and there was neither an internal nor an external validation of the data. In addition, more research is required to investigate the processes that underlie the pathogenic involvement of CLDN4 in OC.

## Figures and Tables

**Table 1:** Association of CLDN4 expression levels with clinical factors in ovarian cancer patients.

Characteristic	Low expression of CLDN4	High expression of CLDN4	*P*
*n*	189	190	
Age, *n* (%)			1.000
≤60	104 (27.4%)	104 (27.4%)	
>60	85 (22.4%)	86 (22.7%)	
FIGO stage, *n* (%)			0.203
Stage I	0 (0%)	1 (0.3%)	
Stage II	14 (3.7%)	9 (2.4%)	
Stage III	150 (39.9%)	145 (38.6%)	
Stage IV	23 (6.1%)	34 (9%)	
Histologic grade, *n* (%)			0.269
G1	1 (0.3%)	0 (0%)	
G2	19 (5.1%)	26 (7%)	
G3	165 (44.7%)	157 (42.5%)	
G4	0 (0%)	1 (0.3%)	
Lymphatic invasion, *n* (%)			0.817
No	23 (15.4%)	25 (16.8%)	
Yes	52 (34.9%)	49 (32.9%)	
Age, median (IQR)	59 (50, 69)	58.5 (52, 67)	0.786

**Table 2:** Univariate and multivariate analysis of overall survival in patients with ovarian cancer.

Characteristics	Total (*N*)	Univariate analysis		Multivariate analysis	
		Hazard ratio (95% CI)	*P* value	Hazard ratio (95% CI)	*P* value
Age	377				
≤60	206	Reference			
>60	171	1.355 (1.046-1.754)	**0.021**	1.410 (1.088-1.828)	**0.009**
FIGO stage	374				
Stage I and stage II	24	Reference			
Stage III and stage IV	350	2.115 (0.938-4.766)	0.071	2.122 (0.942-4.781)	0.070
Histologic grade	367				
G1 and G2	46	Reference			
G3 and G4	321	1.229 (0.830-1.818)	0.303		
Lymphatic invasion	148				
No	48	Reference			
Yes	100	1.413 (0.833-2.396)	0.200		
CLDN4	377				
Low	187	Reference			
High	190	1.647 (1.263-2.147)	**<0.001**	1.693 (1.297-2.209)	**<0.001**

**Table 3:** Univariate and multivariate analysis of disease-specific survival in patients with ovarian cancer.

Characteristics	Total (*N*)	Univariate analysis		Multivariate analysis	
		Hazard ratio (95% CI)	*P* value	Hazard ratio (95% CI)	*P* value
Age	352				
≤60	196	Reference			
>60	156	1.255 (0.950-1.658)	0.110		
FIGO stage	350				
Stage I and stage II	23	Reference			
Stage III and stage IV	327	2.276 (0.935-5.541)	0.070	2.283 (0.938-5.555)	0.069
Histologic grade	342				
G1 and G2	42	Reference			
G3 and G4	300	1.394 (0.893-2.178)	0.144		
Lymphatic invasion	144				
No	48	Reference			
Yes	96	1.397 (0.810-2.408)	0.229		
CLDN4	352				
Low	183	Reference			
High	169	1.554 (1.171-2.063)	**0.002**	1.553 (1.170-2.061)	**0.002**

**Table 4:** Univariate and multivariate analysis of progress-free interval in patients with ovarian cancer.

Characteristics	Total (*N*)	Univariate analysis		Multivariate analysis	
		Hazard ratio (95% CI)	*P* value	Hazard ratio (95% CI)	*P* value
Age	377				
≤60	206	Reference			
>60	171	1.076 (0.848-1.366)	0.547		
FIGO stage	374				
Stage I and stage II	24	Reference			
Stage III and stage IV	350	1.573 (0.918-2.694)	0.099	1.573 (0.918-2.694)	0.099
Histologic grade	367				
G1 and G2	46	Reference			
G3 and G4	321	1.188 (0.835-1.688)	0.338		
Lymphatic invasion	148				
No	48	Reference			
Yes	100	1.115 (0.729-1.704)	0.615		
CLDN4	377				
Low	187	Reference			
High	190	1.183 (0.933-1.500)	0.165		

## Data Availability

The data used to support the findings of this study are available from the corresponding author upon request.

## Supplementary Materials

Supplementary Materials Figure S1: pancancer analysis of CLDN4 based on (A) TCGA datasets or (B) TCGA and GTEx database.
