# Supplementary figures and images for: CLDN4 as a Novel Diagnostic and Prognostic Biomarker and Its Association with Immune Infiltrates in Ovarian Cancer
Source: Mediators Inflamm. 2023 Apr 4;2023:1075265. doi: 10.1155/2023/1075265 (PMC10089777; doi:10.1155/2023/1075265)

**A**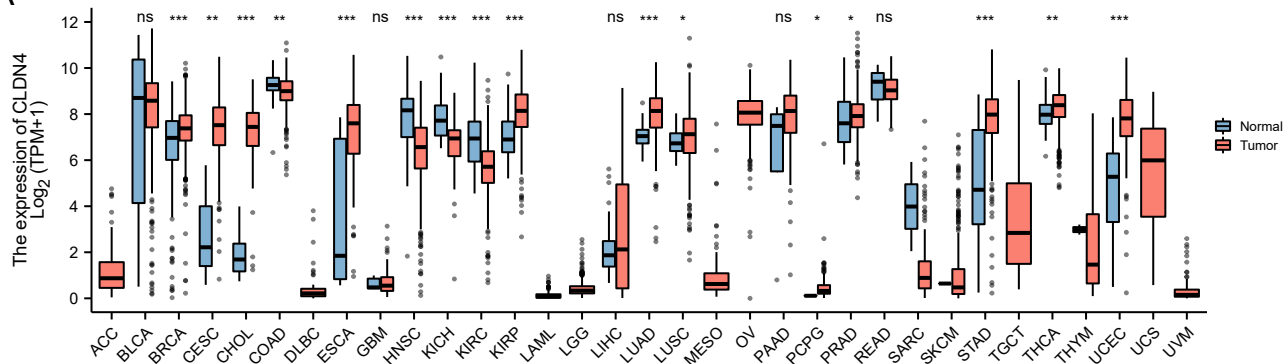**B**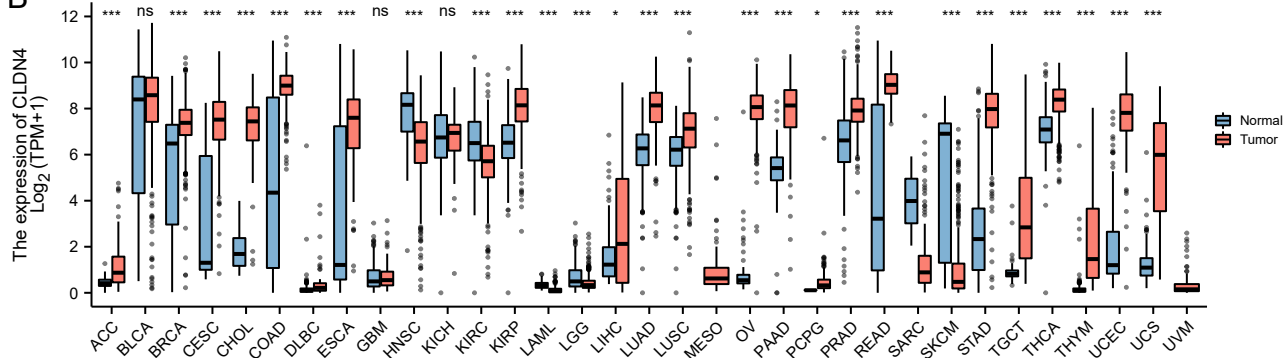

Supplement: Supplementary Materials — Figure S1: pancancer analysis of CLDN4 based on (A) TCGA datasets or (B) TCGA and GTEx database. [file 1075265.f1.pdf]
